# Posterior transpedicular approach with circumferential debridement and anterior reconstruction as a salvage procedure for symptomatic failed vertebroplasty

**DOI:** 10.1186/s13018-015-0169-9

**Published:** 2015-02-10

**Authors:** Yen-Chun Chiu, Shih-Chieh Yang, Hung-Shu Chen, Yu-Hsien Kao, Yuan-Kun Tu

**Affiliations:** Department of Orthopedic Surgery and Anesthesiology, E-Da Hospital/I-Shou University, 1, E-Da Road, Kaohsiung City, Taiwan

**Keywords:** Cement dislodgement, Cement leakage, Failed vertebroplasty, Posterior transpedicular approach, Spinal infection

## Abstract

**Background:**

Complications and failure of vertebroplasty, such as cement dislodgement, cement leakage, or spinal infection, usually result in spinal instability and neural element compression. Combined anterior and posterior approaches are the most common salvage procedure for symptomatic failed vertebroplasty. The purpose of this study is to evaluate the feasibility and efficacy of a single posterior approach technique for the treatment of patients with symptomatic failed vertebroplasty.

**Methods:**

Ten patients with symptomatic failed vertebroplasty underwent circumferential debridement and anterior reconstruction surgery through a single-stage posterior transpedicular approach (PTA) from January 2009 to December 2011 at our institution. The differences of visual analog scale (VAS), neurologic status, and vertebral body reconstruction before and after surgery were recorded. The clinical outcomes of patients were categorized as excellent, good, fair, or poor based on modified Brodsky’s criteria.

**Results:**

The symptomatic failed vertebroplasty occurred between the T11 and L3 vertebrae with one- or two-level involvement. The average VAS score was 8.3 (range, 7 to 9) before surgery, significantly decreased to 3.2 (range, 2 to 4) after surgery (*p* < 0.01), and continued to decrease to 2.4 (range, 2 to 3) 1 year later (*p* < 0.01). The average correction of Cobb’s angle after surgery was 17.3° (range, 4° to 35°) (*p* < 0.01). The mean loss of Cobb’s angle correction after 1 year of follow-up was 2.7° (range, 0° to 5°). The average allograft subsidence at 1 year after surgery was 1 mm (range, 0 to 2). The neurologic status of Frankel’s scale significantly improved after surgery (*p* = 0.014) and at 1 year after surgery (*p* = 0.046). No one experienced severe complications such as deep wound infection or neurologic deterioration. All patients achieved good or excellent outcomes after surgery based on modified Brodsky’s criteria (*p* < 0.01).

**Conclusions:**

Single-stage PTA surgery with circumferential debridement and anterior reconstruction technique provides good clinical outcomes and low complication rate, which can be considered as an alternative method to combined anterior and posterior approaches for patients with symptomatic failed vertebroplasty.

## Background

Percutaneous vertebroplasty (PV) is a minimally invasive procedure which was first developed by Galibert and Deramond for the treatment of hemangioma in 1987 [1]. This technique involves a posterior transpedicular approach (PTA) using a spinal needle under fluoroscopic guidance and injection of polymethyl-methacrylate (PMMA) cement into a collapsed vertebral body. Thereafter, it has become a widespread procedure for management of osteoporotic vertebral compression fracture (VCF), multiple myeloma, lymphoma, and metastatic spinal tumor [2-5].

The use of PV for osteoporotic VCF can achieve significant pain relief and satisfactory clinical outcomes. However, the long-term results remain unpredictable [6-8]. Symptomatic complications and failure of vertebroplasty are uncommon and most of them can be resolved by conservative methods. Surgical intervention is indicated for some circumstances, such as spinal instability and neural element compression due to cement dislodgement, cement leakage, and spinal infection [9]. The standard salvage procedure required a combined surgical technique, which involves anterior transthoracic or retroperitoneal approach for direct debridement and decompression, accompanied by bone grafting or body spacer reconstruction, and posterior approach for supplemental posterior instrumentation and fusion. However, most patients with symptomatic failed vertebroplasty are elderly and have rather poor health status. These patients may not be amenable to an anterior approach due to poor pulmonary function, concurrent medical illness, previous surgery, or previous radiation therapy. Additionally, the disadvantages of combined approaches, including the requirement of prolonged anesthetic and surgical sessions, separated surgical wound, and the need to perform diaphragm takedown and rib cutting, tend to hurt this fragile population [10,11].

In an attempt to decrease the morbidity associated with the combined surgical procedures, a single-stage technique using PTA, with circumferential decompression and reconstruction, has been proposed. Many surgeons have used this technique to treat patients with spinal disorders caused by metastatic tumor, infection, or burst fracture [12-15]. To our knowledge, there is no previous study using this technique as a salvage procedure for complications and failure of vertebroplasty. The purpose of this study is to evaluate the feasibility and efficacy of this single-stage PTA surgery for the treatment of patients with symptomatic failed vertebroplasty. We also examined and compared the clinical outcomes and radiographic findings of these patients before and after surgery.

## Materials and methods

### Patients

The study population comprised 787 consecutive patients who underwent PV for treatment of osteoporotic VCFs from January 2009 to December 2011 at our institution. One-level vertebroplasty was performed in 582 patients, 2 levels in 140, 3 levels in 39, 4 levels in 22, 5 levels in 3, and 6 levels in 1 patient. After a comprehensive review of the medical records, 10 patients who had failed vertebroplasty and underwent a revised single-stage PTA surgery were enrolled in the study. There were 7 women and 3 men with an average age of 77.9 years (range, 69 to 90 years). The patients’ medical records, including outpatient and emergency room notes, admission notes, inpatient progress and nursing notes, discharge summaries, procedure notes, surgical reports, radiology reports, pathology reports, and microbiology laboratory results, were reviewed.

These 10 patients with failed vertebroplasty had symptoms of progressive back pain with or without sciatica, which could not be controlled by conservative treatment. Conservative treatment included pain-killers for back pain, braces for spinal instability, and intravenous antibiotics for pyogenic spondylitis. The single-stage PTA surgery was performed for these patients after failure of the conservative treatment. Radiographic assessment was carried out before and after surgery, at the 3-, 6-, and 12-month visit after discharge and every year thereafter. The patients were followed up at our outpatient department 1 week, 1 month, 3 months, and then every 3 months thereafter. All 10 enrolled patients were followed up for at least 12 months after undergoing the revised single-stage PTA surgery.

### Surgical technique

#### Exposure and posterior stabilization

After induction of general anesthesia, a midline incision from 2 levels above the involved vertebrae to 2 levels below was made to expose the posterior complex. The exact level was identified by intraoperative C-arm fluoroscopy and the exposed level was adjusted accordingly. Transpedicular screws were introduced 2 levels above and 2 levels below the involved vertebrae initially. The devices used for instrumentation included USS and ClickX (Synthes, Solothurn, Switzerland). Then, the laminectomy procedure was started and a rod was used temporarily if spinal instability was found after removal of the posterior complex. Since a bulky rod could influence allograft insertion access and transpedicular debridement, the temporary rod was placed to conduct the screws on the contralateral side of the planned allograft insertion. One rod could maintain adequate stability after extensive posterior complex decompression and prevent injury to the neural elements by any undesired manipulated vibration. The planned insertion site was decided based on the preoperative image survey and neurologic findings. Sometimes, bilateral transpedicular debridement was indicated for extensive debridement. In this situation, we placed another rod on the other side which has been finished of debridement and then released the previously fixed rod to continue contralateral debridement; therefore, decompression of both sides could be achieved.

#### Decompression

Facetectomy was performed above and below the involved vertebrae to expose the involved pedicles and neurologic elements. A plane between the posterior longitudinal ligament and the dura was created by blunt dissection. The circumferential debridement procedure was accomplished using PTA through the route of the interval between the nerve roots. A unilateral approach was used if only one side of the neurologic elements was compressed by the protrusion of the fractured vertebral body or leaked bone cement, and a bilateral approach was used if both sides were involved. Ligation and sacrifice of the nerve root were not necessary because the working space was adequate. Although total corpectomy was feasible using the PTA, subtotal vertebrectomy was usually performed to remove the bone cement fragment as completely as possible and create an adequate space for fibular allograft implantation. The disks above and below the cemented vertebrae were removed to prepare the adjacent endplates. The anterior structures, either the anterior cortex or anterior longitudinal ligament, which provided protection of the anterior vessels, diaphragm, visceral organs, or some vital structures, were preserved in most cases.

#### Anterior column reconstruction

After the neural elements were completely decompressed, we gently distracted the 2 screws above and below the involved vertebrae by using a distractor on the rod. During distraction, we needed to observe the nerve roots and spinal cord tension to prevent traction injury of the neural elements. The length of the bony defect was then measured. An adequate length of freeze-dried fibular allograft was prepared and procured. Multiple drilling using Kirschner wires was performed on both ends of the allograft to facilitate bony incorporation between the fibular allograft and the vertebral body. The fibular allograft was introduced through the route between the nerve roots. After the allograft was well deposited, we released the distraction on the rod and then constructed both rods and a link for immediate stability. Sometimes, we even compressed the adjacent instrumentation to obtain good contact between the endplates and allograft and achieve better spinal alignment.

#### Postoperative care

After surgery, the neurologic status and hemodynamic status were checked regularly. We also administered anti-osteoporosis drugs to every patient to decrease the possibility of screw loosening, fixation failure, and further compression fracture at other levels. Patients were mobilized by a chair or wheelchair on the first postoperative day; ambulation training was started if the pain and neurologic status could be tolerated. Taylor’s brace or body jacket was arranged for protection of the spine. Eight of the patients received prophylactic antibiotics for 2 days. Two patients who underwent surgery due to spinal infection were prescribed for 6 weeks intravenous antibiotics based on culture data. The culture data of both patients showed oxacillin-sensitive *Staphylococcus aureus*. The other 8 patients were discharged within 14 days after surgery and were followed up for at least 1 year at the outpatient department.

### Outcome assessment

Clinical outcomes were assessed by asking the patients to evaluate their pain on a visual analog scale (VAS, using a scale of 0–10; 0 meaning no pain and 10 the most pain possible) and on the basis of pain, activity, and analgesics requirement during admission, before discharge, and at 1 year follow-up to determine the modified Brodsky’s criteria, which were categorized as poor, fair, good, and excellent [16]. The severity of the neurological status was evaluated using the Frankel scale before surgery, at discharge, and 1 year later. The correction of the sagittal Cobb’s angle before surgery was compared with that at discharge and after surgery 1 year later using radiographic image examination. The Cobb’s angle, defined as the angle between the superior endplate of the cephalad-instrumented vertebra and the inferior endplate of the caudal-instrumented vertebra, was measured on plain lateral radiograph. The VAS, modified Brodsky’s criteria, Frankel scale, and sagittal Cobb’s angle before surgery were compared with those after surgery and 1 year later using the Wilcoxon signed-rank test. Nonparametric statistics were used because some variables did not have normally distributed data. SPSS 13.0 software (SPSS Inc., Chicago, USA) was used for data analysis. A value of *P* < 0.05 was considered statistically significant.

## Results

**Figure 1:**
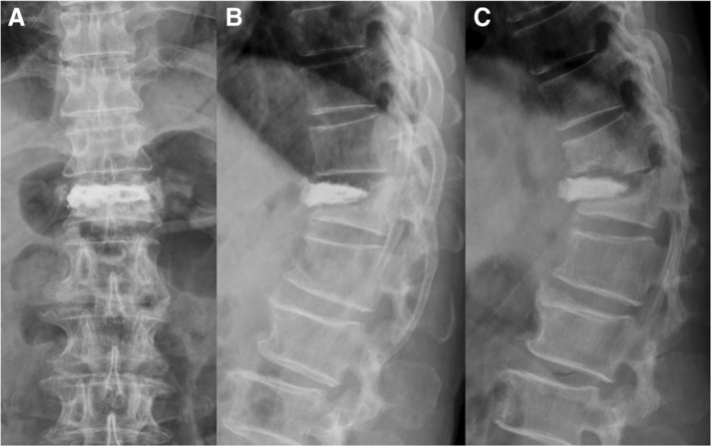
**A 78**-**year**-**old man underwent PV for T12 osteoporotic VCF.** The patient experienced intractable back pain and bilateral lower limb weakness after falling down and injuring himself. Anteroposterior radiograph **(A)** revealed a radiolucent line surrounding the bone cement. Lateral radiograph **(B)** revealed T12 bone cement anterior dislodgement. After 2 months of conservative treatment, the symptoms of back pain and leg weakness had worsened. The follow-up lateral radiograph **(C)** showed further cement dislodgement and T11 adjacent fracture with kyphotic deformity.

**Figure 2:**
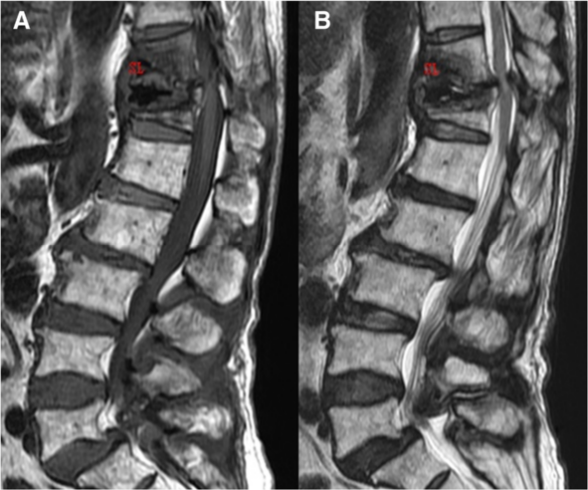
**Sagittal MRI images.** Sagittal T1-weighted MRI **(A)** showed T11 and T12 fracture with bone marrow edema. Sagittal T2-weighted MRI **(B)** revealed fluid accumulation surrounding T12 bone cement. Both sagittal T1- and T2-weighted MRI demonstrated T11 and T12 severe stenosis due to spinal instability.

**Figure 3:**
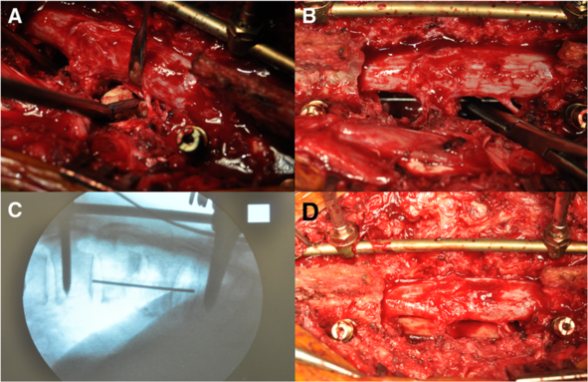
**After adequate debridement through single**-**stage PTA surgery, the dislodged cement was identified and removed.** A rod was temporarily placed on the contralateral side of the planned allograft insertion to prevent undesired vibration during operation **(A)**. A Kirschner wire was used to measure an adequate length for the allograft **(B)**. An intraoperative fluoroscope was used to make sure that the length was suitable. The Kirschner wire should touch the lower endplate of the upper vertebral body and upper endplate of the lower vertebral body **(C)**. After gently retracting the rod, a well-prepared fibular allograft was inserted carefully through the route between the nerve roots. The rods were used to provide stability after adequate compression **(D)**.

**Figure 4:**
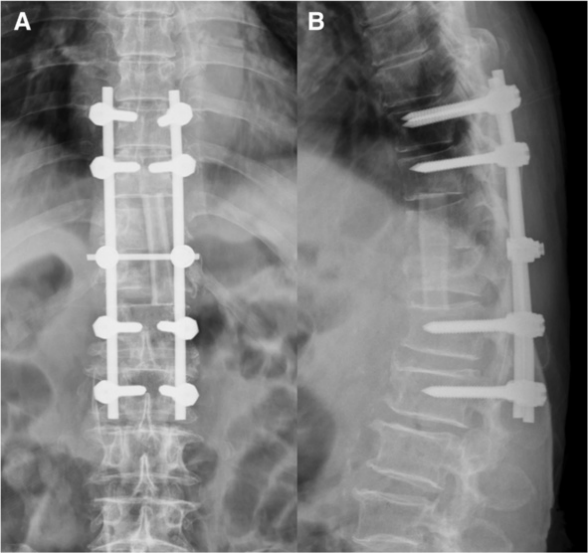
**Postoperative radiographs.** The postoperative anteroposterior **(A)** and lateral **(B)** radiographs revealed that the dislodged bone cement had been removed. Good correction of spinal alignment was achieved by fibular allograft implantation and posterior pedicle screw fixation.

**Figure 5:**
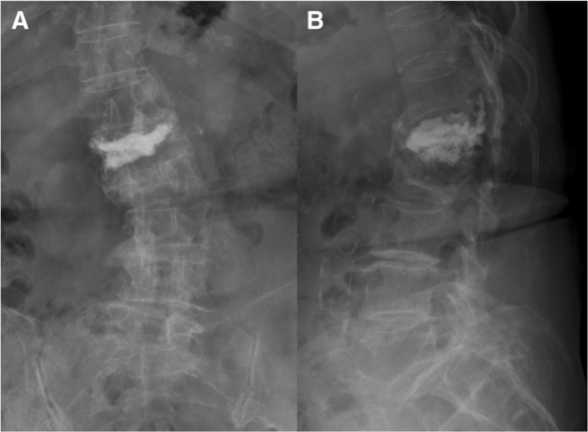
**A 76**-**year**-**old woman who underwent L1 PV sustained progressive intractable back pain with bilateral lower limb numbness and weakness.** The anteroposterior **(A)** and lateral **(B)** radiographs revealed cement leakage into the spinal canal and L2 adjacent fracture.

**Figure 6:**
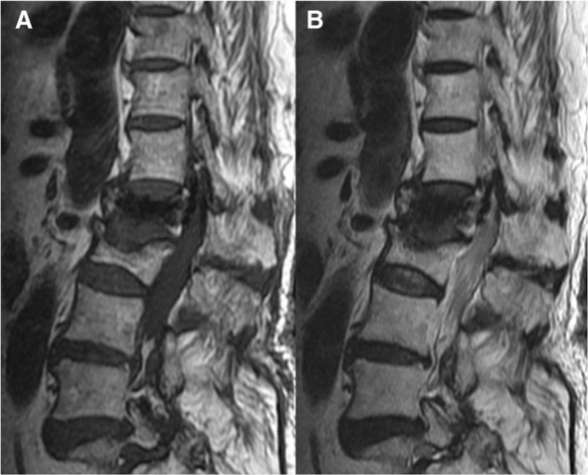
**Sagittal MRI images.** Sagittal T1-weighted MRI **(A)** and sagittal T2-weighted MRI **(B)** revealed bone cement leakage into the spinal canal with neural element compression.

**Figure 7:**
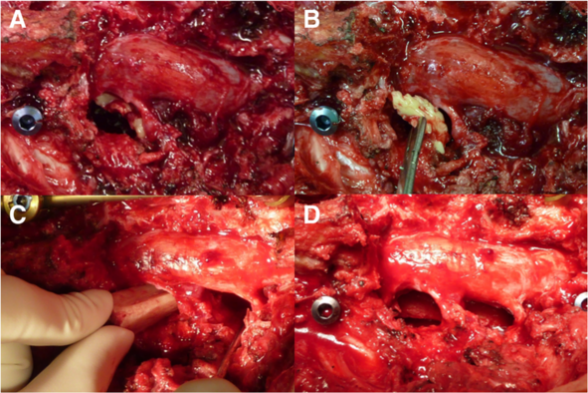
**Cement leakage with neural element compression.** The leaked cement fragment was identified after circumferential decompression through single-stage PTA surgery **(A)**. The cement fragment was removed with a clamp **(B)**. The fibular allograft was inserted through the route between the nerve roots after adequate debridement had been performed **(C)**. The fibular allograft was finally well positioned **(D)**.

**Figure 8:**
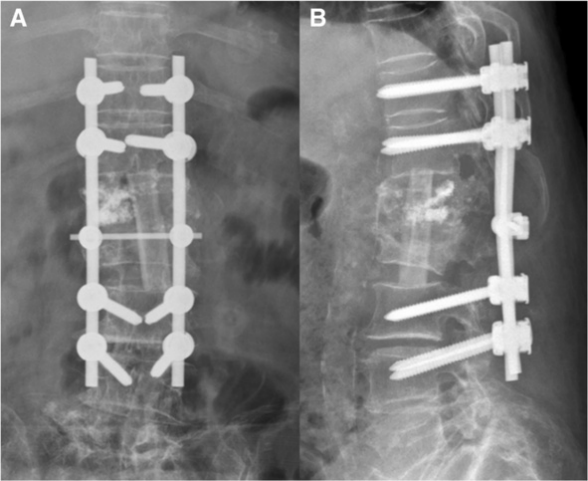
**The postoperative anteroposterior (A) and lateral (B) radiographs revealed the leaked cement fragment within the spinal canal had been removed.** There still was some residual cement with no influence on the neural elements. An acceptable spinal alignment was restored with an adequate length of fibular allograft implantation and posterior pedicle screw fixation.

The average interval between initial vertebroplasties and revised single-stage PTA surgeries was 11.6 months (range, 1 to 25 months). Five patients had symptomatic failed vertebroplasty in 2 levels and 5 in 1 level, and all occurred between the T11 and L3 vertebrae. Six patients underwent instrumentation 2 levels above and 2 levels below the involved vertebrae, and 4 patients underwent instrumentation of extended levels due to the osteoporotic vertebrae. The indications for the revised single-stage PTA surgery for these patients included cement dislodgement in 6 patients (Figures 1, 2, 3, and 4), cement leakage with neural element compression in 2 (Figures 5, 6, 7, and 8), and spinal infection in 2. The average allograft length for reconstruction was 53.8 mm (67.4 mm in 2 failed levels and 40.2 mm in 1 failed level) (Table 1).

**Table 1:** **Patient demographic data**

**Case**	**Age (years)**	**Gender**	**Failed levels**	**Time between PV and surgery (months)**	**Instrumentation level**	**Failed mechanism**	**Allograft length (mm)**
1	76	F	L1 and L2	1	T11T12 to L3L4	Cement leakage	65
2	76	F	T12	12	T10T11 to L1L2	Cement dislodgement	39
3	79	F	L1	5	T10T11 to L3L4	Infection	38
4	89	M	T12 and L1	22	T10T11 to L3L4	Cement dislodgement	69
5	72	F	T11 and T12	1	T7T9 to L2L3	Cement leakage	68
6	71	F	L2 and L3	9	T12L1 to L4L5	Cement dislodgement	72
7	90	M	L3	20	L1L2 to L4L5	Cement dislodgement	52
8	69	F	T12	25	T9T11 to L3L4	Cement dislodgement	37
9	78	M	T11 and T12	18	T9T10 to L1L2	Cement dislodgement	63
10	79	F	L1	3	T11T12 to L2L3	Infection	35

*F* female, *M* male, *L* lumbar spine, *T* thoracic spine, *PV* percutaneous vertebroplasty.

**Figure 9:**
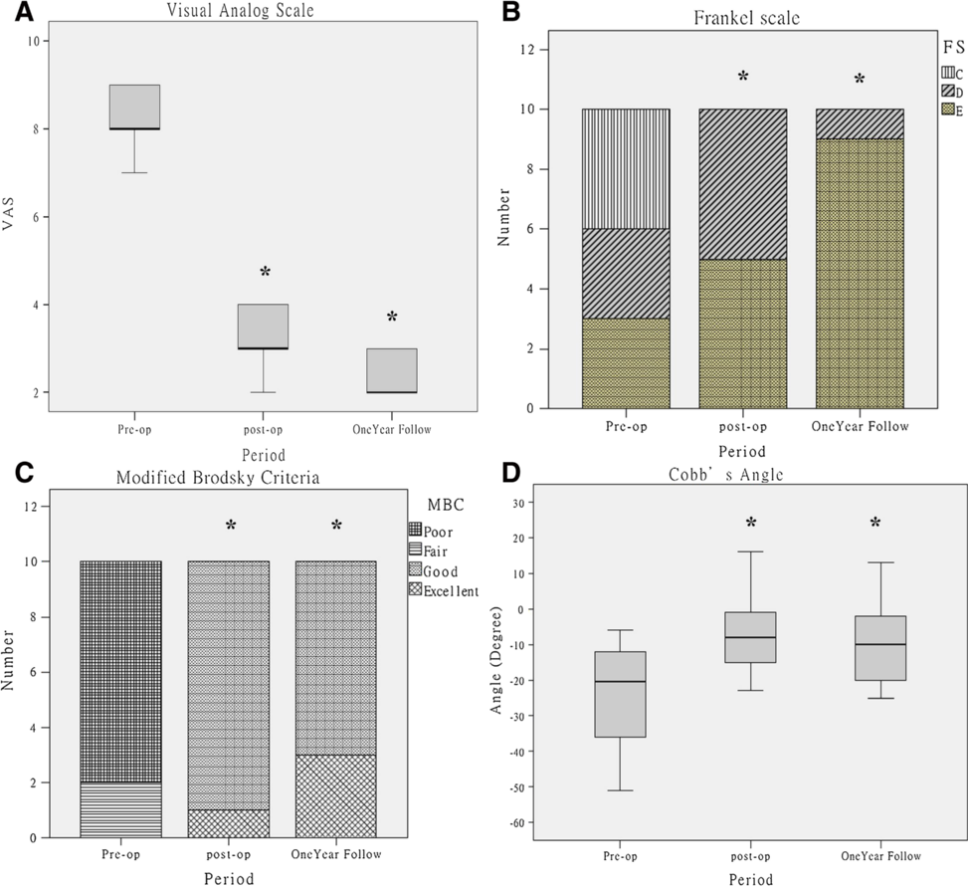
**Comparison of clinical outcomes and radiographic findings before surgery, after surgery, and at the 1**-**year follow**-**up visit.** Visual analog scale **(A)**, Frankel’s scale **(B)**, modified Brodsky’s criteria **(C)**, and Cobb’s angle **(D).**

The most prominent clinical sign of the failed vertebroplasty was severe back pain, which significantly decreased from the average VAS of 8.3 (range, 7 to 9) before surgery to 3.2 (range, 2 to 4) before discharge (*p* < 0.01) and continually decreased to 2.4 (range, 2 to 3) 1 year later (*p* < 0.01) (Figure 9A). Modified Brodsky’s criteria of all patients significantly increased from either poor or fair before surgery to good or excellent at discharge (*p* < 0.01) and 1 year later (*p* < 0.01) (Figure 9C). Neurologic status significantly improved, from the median of Frankel D before surgery to Frankel E before discharge (*p* = 0.014) and at the 1-year follow-up (*p* = 0.015) (Figure 9B). Only 5 patients still had abnormal neurologic function with Frankel D before discharge, and 4 of them improved to normal function 1 year later. No patients experienced neurologic deterioration after surgery (Table 2).

**Table 2:** **The improvement of visual analog scale, Frankel’s scale, and modified Brodsky’s criteria before surgery, after surgery, and 1 year later**

**Case number**	**Preop VAS**	**Postop VAS**	**1 yr VAS**	**Preop MBC**	**Postop MBC**	**1 yr MBC**	**Preop FS**	**Postop FS**	**1 yr FS**
1	9	3	2	P	G	G	C	D	E
2	7	3	3	F	E	E	E	E	E
3	8	2	2	P	G	E	C	D	E
4	9	4	3	P	G	G	D	D	E
5	8	4	3	P	G	G	C	D	D
6	8	3	2	P	G	G	D	E	E
7	9	3	2	P	G	E	D	E	E
8	8	4	3	P	G	G	E	E	E
9	9	3	2	P	G	G	C	D	E
10	8	3	2	F	G	G	E	E	E

*Preop* preoperative; *Postop* postoperative; *1 yr* 1 year later; *VAS* visual analog scale: 0 means no pain and 10 means the most pain possible; *MBC* modified Brodsky’s criteria: *P* poor, *F* fair, *G* good, *E* excellent; *FS* Frankel’s scale: *A* complete paralysis, *B* sensory function only below the injury level, *C* incomplete motor function below injury level, *D* fair to good motor function below injury level, *E* normal function.

The sagittal Cobb’s angle was significantly corrected, from a mean angle of −23.3° (range, −51° to −6°) before surgery to −6.0° (range, −23° to 16°) after surgery (*p* < 0.01) and −8.7° (range, −25° to 13°) 1 year later (*p* < 0.01) (Figure 9D). Kyphotic deformity improved in all patients, with an average angle correction of 17.3° (range, 4° to 35°) immediately after surgery. One year later, the mean loss of Cobb’s angle correction was 2.7° (range, 0° to 5°), and no bone graft dislodgement or screw loosening was found (Table 3). Surgery-related complications such as cerebrospinal fluid leakage, wound infection, pseudoarthrosis, nonunion, and loss of fixation were not encountered in our patient series during at least 1 year of follow-up.

**Table 3:** **The changes of radiographic findings before and after surgery**

**Case number**	**Preop Cobb’s angle (°)** ^**a**^	**Postop Cobb’s angle (°)** ^**a**^	**Correction of Cobb’s angle (°)**	**Loss of correction at 1 year (°)**	**Allograft subsidence (mm)**
1	−10	−1	9	1	1
2	−21	−7	14	0	0
3	−13	−9	4	2	1
4	−39	−23	16	2	1
5	−12	−5	7	4	1
6	−20	15	35	5	2
7	−6	16	22	3	1
8	−36	−15	21	5	2
9	−51	−22	29	2	1
10	−25	−9	16	3	1

*Preop* preoperative, *Postop* postoperative.

^a^Sagittal kyphotic angle (opposed to sagittal lordotic angle).

## Discussion

Treatment of complications and failure of vertebroplasty is a challenge for spinal surgeons because the proper salvage method for this complicated condition is still controversial [9,17]. A combined anterior and posterior surgery has been reported to be the most secure method. However, anterior approach to the spine, especially the upper thoracic and lower lumbar spine, is technically difficult and highly dependent on surgical experience. PTA with circumferential decompression and anterior reconstruction was developed to manage complicated spinal disorders, such as spinal tumor, infection, and burst fracture. Good clinical outcomes and low complication rate have been mentioned in the literature [12-15]. To our knowledge, the current study is the first report using this unique technique as a salvage procedure to treat patients with symptomatic failed vertebroplasty.

Six patients in this study underwent single-stage PTA surgery due to cement dislodgement resulting in spinal instability and associated stenosis. A proposed mechanism of this complication is a re-fracture of the cemented vertebrae at the stress junction of cement and bone or the posterior cortex defect, which allows relative motion of the cement block and results in final displacement with a further bending load [18]. The potential risk for cement dislodgment is an intravertebral vacuum cleft caused by avascular necrosis preoperatively [19]. Injection of PMMA into a cystic cavity would be expected to have far less interdigitation with the surrounding bone than injection into partially intact trabecular bone. The fluid in an intraosseous vacuum may isolate the cement filling in the fractures around it [20]. Therefore, PMMA cement may be only a space-occupying material without mechanical interlock and biocompatibility. As a result, further neural element irritation may occur due to direct compression by the posterior displaced bone cement and/or reactive stenosis by the hypertrophic facet joints and ligamentum flavum.

The complications related to cement leakage during vertebroplasties have been extensively discussed in the literature. Most of these complications are transient and minor in severity [21,22]. Cement leakage into the spinal canal frequently occurs with the presence of posterior vertebral cortex destruction. Cement leakage may be well tolerated if there is sufficient residual space for the spinal cord, but it could be a disaster if the spilled cement compresses the spinal cord and/or nerve roots, resulting in neurologic deficit [23,24]. Urgent decompression is indicated for these patients and the outcomes usually vary. In a retrospective study, Patel et al. reviewed the clinical course of 14 patients with documented loss of neurologic function following percutaneous vertebral cement augmentation. Six patients developed neurologic deficits acutely (less than 24 h), and the remaining 8 patients developed neurologic symptoms at an average of 37.1 days (range, 3–112 days). Twelve of 14 patients (85.7%) required revision open surgery for treatment of their neurologic injury [25]. Two patients with neurologic deficits due to cement leakage into the spinal canal were included in our study. Progressive onset of neurologic disorders may result from leaked bone cement and associated spinal instability. Single-stage PTA surgery allowed these patients early mobilization and rehabilitation regardless of their neurologic status, therefore yielding good functional recovery and decreasing overall morbidity and mortality.

Spinal infection is uncommon after vertebroplasties [26]. Most of these patients can be treated successfully with conservative methods. There are four main indications for surgery: failure of a CT-guided biopsy or blood culture to yield an organism, thus necessitating open or percutaneous retrieval of more tissue; failure of medical therapy; development of neurological disorder; and structural decompensation. The algorithm for surgical treatment requires considering four goals of management: thorough radical debridement, relieving pressure on neural elements, restoring normal alignment, and providing rigid fixation. The anterior approach is favored by many surgeons because of the direct assessment of pathogen debridement and accompanied reconstruction of the anterior column. Although it could be an effective method, significant morbidity, including vascular, visceral, or pulmonary complications, is still a major concern for this procedure [27]. In a clinical study, 85 patients were treated using an anterolateral transthoracic approach for various lesions of the thoracic and thoracolumbar spine. The reported rate of severe approach-related complications occurred in 4 patients (4.7%) [28]. The anterior approach is also poorly tolerated by patients with poor pulmonary function because lung deflation is needed during this approach. Visocchi et al. have proposed that if preoperative evaluation shows a partial pressure of oxygen (*P*_O2_) of less than 60 mmHg, partial pressure of carbon dioxide (*P*co_2_) of more than 45 mmHg, oxygen (O_2_) saturation below 90%, forced vital capacity (FVC) less than 1.5 L, forced expiratory volume in 1 s (FEV_1_) less than 1 L, and FEV_1_/FVC less than 35%, the transthoracic approach is contraindicated [29]. Most patients undergoing vertebroplasty are elderly and have other comorbidities. With these coexisting underlying diseases, the anterior approach becomes risky for these patients. Additionally, most vertebroplasties for osteoporotic VCFs are performed at the thoracolumbar junction. A diaphragm takedown procedure is necessary if the anterior approach is used for lesions at the thoracolumbar junction, and this may lead to pulmonary function impairment after surgery.

Nerve root ligation and sacrifice to create a wider working space are common during the procedure of posterior approach at thoracic levels. However, rhizotomy may result in dysesthetic pain syndrome. Different from costotransversectomy or lateral extracavitary approach, PTA provides a safe corridor to access the anterior thoracolumbar column. Circumferential debridement, including bone cement removal and partial corpectomy, can be achieved piece by piece using clamps or rongeurs, through the route between the nerve roots. Therefore, the nerve roots can be intact preserved. Additionally, the space is also adequate for fibular allograft implantation. A corpectomy defect was traditionally reconstructed with structural autologous bone grafts from the iliac crest or fibula, which may cause an increase in permanent donor site morbidity up to 25% [30,31]. The concern about donor site-related complications has led the authors to use allogenous bone grafts for the reconstruction. An ideal graft construct should have the ability of bony incorporation without collapse and with the fewest undesired complications. A cortical graft such as a cadaveric humerus or fibula, which has a much higher modulus of elasticity and is less prone to collapse acutely over time, would be a good choice [32,33].

## Conclusions

Single-stage PTA surgery can simultaneously accomplish circumferential decompression, fibular allograft anterior implantation, and supplemental posterior instrumentation to salvage patients with symptomatic failed vertebroplasty. Good clinical outcomes and low complication rate can be achieved by this unique technique. Elderly patients usually cannot tolerate the prolonged and complex surgery. Therefore, single-stage PTA surgery could be specially considered as an alternative method to combined anterior and posterior surgeries for these fragile patients.

## Abbreviations

PV: Percutaneous vertebroplasty
PMMA: Polymethyl-methacrylate
VCF: Vertebral compression fracture
PTA: Posterior transpedicular approach
VAS: Visual analog scale
FVC: Forced vital capacity
FEV1: Forced expiratory volume in 1 s
